# Antihypertensive Drug Recommendations for Reducing Arterial Stiffness in Patients With Hypertension: Machine Learning–Based Multicohort (RIGIPREV) Study

**DOI:** 10.2196/54357

**Published:** 2024-11-25

**Authors:** Iván Cavero-Redondo, Arturo Martinez-Rodrigo, Alicia Saz-Lara, Nerea Moreno-Herraiz, Veronica Casado-Vicente, Leticia Gomez-Sanchez, Luis Garcia-Ortiz, Manuel A Gomez-Marcos, RIGIPREV Investigators Group

**Affiliations:** 1 CarVasCare Research Group Facultad de Enfermería de Cuenca Universidad de Castilla-La Mancha Cuenca Spain; 2 Facultad de Ciencias de la Salud Universidad Autónoma de Chile Talca Chile; 3 Department of Informatic Systems University of Castilla‐La Mancha Cuenca Spain; 4 Parquesol University Health Centre West Valladolid Primary Healthcare Management Castilla y León Regional Health Authority Valladolid Spain; 5 Department of Medicine, Dermatology and Toxicology University of Valladolid Valladolid Spain; 6 Emergency Service University Hospital of La Paz Madrid Spain; 7 Primary Care Research Unit of Salamanca Salamanca Primary Healthcare Management Institute of Biomedical Research of Salamanca Salamanca Spain; 8 Research Network on Chronicity Primary Care and Health Promotion Salamanca Spain; 9 Department of Biomedical and Diagnostic Sciences University of Salamanca Salamanca Spain; 10 Department of Medicine University of Salamanca Salamanca Spain; 11 see Acknowledgements

**Keywords:** antihypertensive, drugs, models, patients, pulse wave velocity, recommendations, hypertension, machine learning, drug recommendations, arterial stiffness, RIGIPREV

## Abstract

**Background:**

High systolic blood pressure is one of the leading global risk factors for mortality, contributing significantly to cardiovascular diseases. Despite advances in treatment, a large proportion of patients with hypertension do not achieve optimal blood pressure control. Arterial stiffness (AS), measured by pulse wave velocity (PWV), is an independent predictor of cardiovascular events and overall mortality. Various antihypertensive drugs exhibit differential effects on PWV, but the extent to which these effects vary depending on individual patient characteristics is not well understood. Given the complexity of selecting the most appropriate antihypertensive medication for reducing PWV, machine learning (ML) techniques offer an opportunity to improve personalized treatment recommendations.

**Objective:**

This study aims to develop an ML model that provides personalized recommendations for antihypertensive medications aimed at reducing PWV. The model considers individual patient characteristics, such as demographic factors, clinical data, and cardiovascular measurements, to identify the most suitable antihypertensive agent for improving AS.

**Methods:**

This study, known as the RIGIPREV study, used data from the EVA, LOD-DIABETES, and EVIDENT studies involving individuals with hypertension with baseline and follow-up measurements. Antihypertensive drugs were grouped into classes such as angiotensin-converting enzyme inhibitors (ACEIs), angiotensin receptor blockers (ARBs), β-blockers, diuretics, and combinations of diuretics with ACEIs or ARBs. The primary outcomes were carotid-femoral and brachial-ankle PWV, while the secondary outcomes included various cardiovascular, anthropometric, and biochemical parameters. A multioutput regressor using 6 random forest models was used to predict the impact of each antihypertensive class on PWV reduction. Model performance was evaluated using the coefficient of determination (*R*^2^) and mean squared error.

**Results:**

The random forest models exhibited strong predictive capabilities, with internal validation yielding *R*^2^ values between 0.61 and 0.74, while external validation showed a range of 0.26 to 0.46. The mean squared values ranged from 0.08 to 0.22 for internal validation and from 0.29 to 0.45 for external validation. Variable importance analysis revealed that glycated hemoglobin and weight were the most critical predictors for ACEIs, while carotid-femoral PWV and total cholesterol were key variables for ARBs. The decision tree model achieved an accuracy of 84.02% in identifying the most suitable antihypertensive drug based on individual patient characteristics. Furthermore, the system’s recommendations for ARBs matched 55.3% of patients’ original prescriptions.

**Conclusions:**

This study demonstrates the utility of ML techniques in providing personalized treatment recommendations for antihypertensive therapy. By accounting for individual patient characteristics, the model improves the selection of drugs that control blood pressure and reduce AS. These findings could significantly aid clinicians in optimizing hypertension management and reducing cardiovascular risk. However, further studies with larger and more diverse populations are necessary to validate these results and extend the model’s applicability.

## Introduction

### Background

According to the Global Burden of Diseases, Injuries, and Risk Factors Study, the leading global risk factor for attributable deaths in 2019 was high systolic blood pressure, accounting for 10.8 million deaths (19.2% of the total) [1]. However, despite receiving treatment through both nonpharmacological and pharmacological measures, a significant percentage of patients continue to have poor blood pressure control [2]. Current clinical practice guidelines recommend individualized regimens for hypertension treatment [3,4]. In addition, arterial stiffness (AS), assessed through pulse wave velocity (PWV), is an independent cardiovascular risk factor with the capacity to predict morbidity and mortality from cardiovascular diseases [5,6]. Furthermore, there are numerous pharmacological strategies for hypertension treatment [7], and the effect on AS varies among these groups [8-12]. In a recently published network meta-analysis by our group [8] focusing on the outcomes of the 5 classes of drugs used in clinical practice for hypertension treatment, a significant decrease in PWV was observed with angiotensin-converting enzyme inhibitors (ACEIs), angiotensin receptor blockers (ARBs), and β-blockers (BBs) [8]. The meta-analysis conducted by Ong et al [12] compared the effects of antihypertensive drugs (except ARBs) with those of placebo and revealed that all antihypertensive drugs reduced the PWV. In short-term trials, ACEIs were more effective than calcium channel blockers (CCBs), and in long-term trials, ACEIs, CCBs, BBs, and diuretics were more effective than placebo. The meta-analysis conducted by Chen et al [11] analyzed the effects of ARBs versus other antihypertensive agents on PWV reduction and found no differences compared to other groups. The meta-analysis conducted by Shahin et al [9] examined the effect of ACEIs on AS compared to that of placebo or other antihypertensive agents and concluded that ACEIs reduce PWV but did not definitively establish whether the effect was superior to that of other antihypertensive agents. Finally, the meta-analysis conducted by Li et al [10] assessed the effect of ACEIs on PWV without finding any differences compared to other antihypertensive drugs but did find differences compared to placebo. Similarly, longitudinal studies that have analyzed the effects of ACEIs or ARBs alone or in combination with CCBs or diuretics have demonstrated their effectiveness in reducing PWV [13]. Therefore, all the results suggest that antihypertensive agents may have beneficial effects on AS, but the intensity of these effects may vary. However, no studies have analyzed whether the effects of antihypertensive drugs on AS differ based on the individual characteristics of patients with hypertension.

PWV is now recognized as a crucial indicator of AS, making it a valuable predictor marker for atherosclerosis [14]. This link between hypertension and cardiovascular risk is supported by a strong body of evidence [15]. This study highlights the importance of incorporating PWV into cardiovascular research and risk assessment. Several epidemiological studies, including the well-known Framingham Heart Study [16] and the Rotherdam study [17], have consistently shown a significant link between high PWV and a greater risk of cardiovascular illness and death [5,18,19]. This relationship is further supported by long-term studies that have linked PWV measurements to the occurrence of negative cardiovascular events, such as coronary artery disease and stroke [20]. Selecting PWV as the primary outcome measure provides a thorough understanding of the effects of antihypertensives on arterial health in line with our goal of reducing cardiovascular risk in patients with hypertension [8].

Machine learning (ML) techniques are ubiquitous in all fields of research, and their use in medicine is increasingly common, serving as tools to facilitate diagnosis, prediction, classification, or personalized recommendations based on individual patient characteristics. Unlike rule-based algorithms that require explicit programming, ML algorithms use self-learning techniques to discover sophisticated patterns in medical data, with the potential to enhance personalized care [21]. Therefore, predictive analyses using ML methods are highly valued and practiced in real health care applications, with significant potential to develop recommendations that assist clinicians in making the most suitable decisions for each patient, as the complexity of medicine now exceeds the capacity of the human mind [21,22]. ML techniques have been widely used to predict cardiovascular diseases and their cardiovascular risk factors [23,24]. Numerous studies concerning hypertension have been conducted to predict which antihypertensive drug may be most beneficial for each patient with hypertension [25], analyze the profiles of different antihypertensive drugs [26], build a prediction model for antihypertensive drugs to be used in older patients with hypertension [27], analyze variables influencing blood pressure reduction [28], study variables predicting the risk of hypokalemia in patients with hypertension [29], or predict the onset of pregnancy-induced hypertension [30]. There are many ML studies investigating different personalized recommendations for patients, particularly reinforcement learning algorithms (ie, optimal dosing of medication [31,32] and optimal timing of intervention [33]). However, it is essential to consider the conclusion of the review conducted by the Journal of the American Heart Association in 2023 [22] regarding ML research in patients with hypertension, emphasizing that these ML techniques currently have significant deficiencies in terms of reporting quality, model validation, and bias in the algorithms used.

### Objectives

The hypothesis of this study was that the effects of different antihypertensive drugs on PWV vary according to the individual characteristics of each person with hypertension. To the best of our knowledge, no study has analyzed which antihypertensive drug is most suitable for real clinical practice using ML techniques to reduce PWV. Therefore, we propose this study, with the main objective of developing a model to provide recommendations on the most suitable antihypertensive agent for reducing PWV, based on the individual characteristics of people with hypertension, using advanced ML techniques consisting of multiple random forest (RF) models within a multioutput regressor approach.

## Methods

### Design and Sample Characteristics

The Effectiveness of Antihypertensive Drugs on Arterial Stiffness in Hypertensive Adults Study (the RIGIPREV study) [34] represents a real-world longitudinal investigation involving participants with hypertension. This study drew upon data originating from the Influence of Different Risk Factors in Vascular Accelerated Aging study (the EVA study) [35] (NCT02623894), the Relationship of Central Blood Pressure and Pulse Wave Velocity With Target Organ Damage study (the LOD-DIABETES study) [36] (NCT01065155), and the Effectiveness of the Use of a Mobile Tool in Improving Lifestyle (the EVIDENT study) [37] (NCT02016014). Specifically, participants were selected from these 3 research endeavors if they were using antihypertensive drugs and possessed both baseline and follow-up measurements of variables essential for our primary analysis.

### Ethical Considerations

The research protocols for the studies included in this pooled analysis were approved by the Drug Research Ethics Committee of Salamanca, with the following registration numbers: PI15/01039 and PI20/10569 (EVA study [35]), PI15/11/2015 (LOD-DIABETES study [36]), and PI83/06/2018 (EVIDENT study [37]). All the participants who participated in these studies provided written informed consent. During the development of the study, the principles of the Declaration of Helsinki [38] and the World Health Organization standards for observational studies were followed. The confidentiality of the participants included was always guaranteed in accordance with the provisions of Organic Law 3/2018, of 5 December, on Personal Data Protection and Guarantee of Digital Rights and Regulation (European Union) 2016/679 of the European Parliament and of the Council of 27 April 2016 , on Data Protection.

### Intervention

The antihypertensive drugs used in the 3 studies were categorized into the following groups: BBs, diuretics, ACEIs, ARBs, and combinations of diuretics+ACEIs and diuretics+ARBs. While other antihypertensive agents from various groups (such as CCBs, α-adrenergic receptor antagonists, centrally acting agents, or direct-acting vasodilators) were used in these studies, they were excluded from our analysis due to their limited representation, each having samples of <10 participants (Textbox 1).

**Antihypertensive groups included in the study.**

**Antihypertensive groups**

- β-blockers include acebutolol, atenolol, atenolol, betaxolol, bisoprolol, carteolol, esmolol, metoprolol, nadolol, oxprenolol, penbutolol, propranolol, timolol, celiprolol, carvedilol, labetalol, nebivolol, and pindolol.
- Diuretics include furosemide, bumetanide, torsemide, bendroflumethiazide, chlorothiazide, chlorthalidone, hydrochlorothiazide, indapamide, polythiazide, trichlormethiazide, amiloride, eplerenone, spironolactone, and triamterene.
- Angiotensin-converting enzyme inhibitors (ACEIs) include benazepril, captopril, cilazapril, enalapril, fosinopril, imidapril, lisinopril, moexipril, perindopril, quinapril, ramipril, trandolapril, and zofenopril.
- Angiotensin receptor blockers (ARBs) include candesartan, eprosartan, irbesartan, losartan, olmesartan, telmisartan, and valsartan.
- Combinations of diuretic+ACEI and diuretic+ARB: these categories involve combinations of medications from the diuretic, ACEI, and ARB classes.

### Outcome

Our primary outcome measure centered on the change in carotid-femoral PWV (cfPWV) and brachial-ankle PWV (baPWV). These measurements were consistently collected across the 3 included studies, following their respective study protocols outlined elsewhere [35-37]. To facilitate comparison, we standardized these 2 types of PWV using normalized values.

Specifically, the following primary outcome measures were measured: the cfPWW was estimated using a SphygmoCor device (AtCor Medical Pty Ltd). By analyzing the carotid and femoral artery pulse waves in the supine position, the time delay was estimated and compared to that of the electrocardiogram R wave, and the cfPWV was calculated. Distances were measured with a tape measure from the sternal notch to the point where the sensor was placed in the carotid and femoral arteries [39]. The baPWV was estimated using a VaSera VS-1500 device (Fukuda Denshi Co Ltd) according to the manufacturer’s instructions. The baPWV was estimated using the following equation: baPWV = ({0.5934×height (cm)+14.4724})/tba, where tba is the time interval between the arm and ankle waves [39].

### Covariates

The variables collected and tests performed have been previously published in the protocol of the EVA study [35], the LOD-DIABETES study [36], and the EVIDENT II study [37]. The professionals who performed the tests and questionnaires followed a standardized protocol.

In this study, we meticulously considered an extensive array of covariates, each of which played a crucial role in shaping our analysis. These covariates included demographic factors such as age and sex; lifestyle characteristics such as smoking habits and alcohol consumption; adherence to the Mediterranean diet assessed with the Mediterranean Diet Adherence Screener questionnaire [40]; and medication use, which included antidiabetics, antiaggregants, anticlotting agents, and nonsteroidal anti-inflammatory drugs.

Furthermore, we incorporated a comprehensive set of anthropometric measurements, including height, weight, and waist circumference, along with vital cardiovascular parameters such as systolic and diastolic blood pressure and heart rate, and the measurements were performed according to the recommendations of the European Society of Hypertension [41]. Furthermore, we included parameters such as the ankle-brachial index, intima-media thickness, carotid-artery vascular index (CAVI), and augmentation index at 75 mm Hg (AIx75), ensuring that our statistical models were comprehensive and robust, thereby enhancing the validity of our findings. In addition, the Cornell index and left ventricular hypertrophy were measured.

We also included numerous biochemical variables. The participants in the 3 studies underwent a venous blood draw at the Salamanca Primary Care Research Unit between 8 AM and 9 AM on an empty stomach without smoking or consuming alcohol or caffeinated beverages during the previous 12 hours, and all samples were analyzed in the same laboratory. Specifically, we performed the following analytical determinations (Textbox 2).

**Analytical determinations performed in the study.**

**Analytical determinations**

- Hematological variables, including red blood cells, hemoglobin, mean corpuscular hemoglobin concentration, mean corpuscular hemoglobin content, and mean corpuscular volume, were collected. Variables related to the distribution of erythrocyte sizes, such as the anisocytosis index, were also considered. Moreover, leukocyte subpopulations, including neutrophils, lymphocytes, monocytes, eosinophils, and basophils, red blood cell distribution width, along with platelet characteristics such as the mean platelet volume, were included in our covariate set.
- Regarding diabetes-related covariates, we incorporated fasting plasma glucose, glycated hemoglobin, and insulin levels as essential factors.
- In addition, lipid profiles, comprising total cholesterol, low-density lipoprotein cholesterol, high-density lipoprotein cholesterol, and triglycerides, were considered.
- Renal function markers such as uric acid, serum creatinine, and creatinine, as well as microalbuminuria, reflect kidney health. In addition, the albumin/creatinine ratio and estimated glomerular filtration rate according to the Chronic Kidney Disease Epidemiology Collaboration criteria were included.
- Liver function was assessed through markers, including aspartate aminotransferase and glutamic oxaloacetic transaminase, alanine aminotransferase and serum glutamic pyruvic transaminase, and gamma-glutamyl transferase.
- Inflammation markers, such as fibrinogen and C-reactive protein, were integrated into our covariate framework. Thyroid function was evaluated with the inclusion of thyroid-stimulating hormone and thyroxine levels, while vitamin D levels provided insight into nutritional status.

### Data Standardization

The EVA [35], LOD-DIABETES [36], and EVIDENT [37] studies each followed unique protocols and meticulously selected participants based on predetermined criteria.

To achieve data standardization, our main priority was to ensure consistent measurement of key factors, particularly baseline and follow-up PWV assessments, which were integral to our primary analytical framework. It should be acknowledged that there was no universally agreed upon protocol for drug choice among the 3 studies, but for standardization, we categorized antihypertensive drugs into specific classes, including BB, diuretics, ACEI, ARB, and diuretic+ACEI and diuretic+ARB combinations. Due to their minimal representation within each group, certain medications were excluded from consideration. This was done to ensure substantial sample sizes for meaningful comparisons between groups, a crucial factor in our decision-making process. In addition, patients who consistently stayed on 1 drug throughout the follow-up period as well as those who may have switched between different drugs were included in the analysis.

This study investigated a wide range of antihypertensive drugs that were purposefully selected to enhance the scope and diversity of our research. We acknowledged the diversity and potential differences in drug options and how they are administered in various studies and thus used thorough statistical corrections. Our analysis considered factors such as age, lifestyle behaviors, and medication use, which were carefully incorporated to mitigate any potential biases stemming from these differences.

### Statistical Analysis

Normal probability plots and the Kolmogorov-Smirnov test were used to verify the normality of the distribution of continuous variables. Descriptive data for the total sample and antihypertensive drugs are shown as the means and SDs or proportions (%), as appropriate. ANOVA for continuous variables and chi-square analysis for categorical variables were used to compare baseline variables according to antihypertensive drugs.

### Data Preprocessing and Outcome Structuring

Once the patient data were organized, we addressed missing values within the feature set by imputing them using the median of the respective variables. For the target variables, we created 6 distinct output vectors, each corresponding to 1 of the 6 antihypertensive drugs under study: ACEIs, ARBs, BBs, diuretic, diuretic+ACEI, and diuretic+ARB. These output vectors capture the differences between the original PWV measurements and the PWV values observed during at least 1 year of follow-up for each patient.

### Training and Assessment of Embedded RF ML Models for Multitarget Prediction

To predict medication effectiveness in decreasing PWV, we used a multioutput regressor framework consisting of 6 individual RF models, each corresponding to a different medication [42]. These RF models were independently trained on the same feature set but aimed to predict different target variables, specifically the effectiveness of the respective antihypertensive drugs in decreasing PWV.

To construct the predictive models, we characterized each patient using a feature input vector X, comprising medical attributes such as cholesterol levels, systolic blood pressure, and Mediterranean diet intake, among others. Nevertheless, given the high number of input variables in our study, various procedures for variable reduction were implemented. Initially, the average decrease in impurity for each model was calculated because of the inherent ability of RF algorithms to assess the importance of input features. This analysis was based on evaluating how each covariate contributes to reducing variance in the predictions within the trees. A feature is considered more important if its inclusion in tree splits leads to a greater reduction in variance, indicating that the predictions become more homogeneous and precise due to that feature. In addition, a permutation-based analysis was also conducted to address the potential high cardinality of the input variables, yielding consistent results without unfairly favoring features with a high number of unique values.

Both variable reduction methodologies yielded similar results, leading to the identification of the top 10 most important features for each model and aiming to make the final recommendation system lighter and more interpretable. Thus, each RF model was finally trained using these 10 most important features exclusively in each case. Consequently, the global features used by the system were reduced from 64 to 38 variables, representing a reduction of >40%.

Initially, we combined the 3 studies and opted for an internal validation scheme. Thus, a holdout validation strategy was implemented, randomly distributing data into 2 folds for training and testing, with 80% of patients retained for training [43]. However, we also proceeded with an external validation phase to further test the model’s robustness and generalizability across different datasets. However, given the limited sample size across the 3 databases, the synthetic minority oversampling technique (SMOTE) was used. The SMOTE algorithm works by synthetically generating new samples by selecting instances that are close to the feature space, drawing a line between the examples in the feature space, and creating a new instance at a point along the line [44].

Thus, considering the 3 studies that are part of this work, the samples from the EVIDENT II study were reserved for external validation because they contained the greatest proportion of samples in almost all the subgroups. Meanwhile, the remaining 2 databases were combined and synthetically augmented using the SMOTE algorithm to use the samples for training. These samples were augmented to approximately 80%-20% for training and testing, respectively.

The predictive power of the models was assessed on the validation set in both strategies using the coefficient of determination (*R*^2^) and the mean squared error (MSE) [45]. *R*^2^ quantifies the proportion of the variance in the dependent variable, PWV, predicted from the independent variables, with values closer to 1 indicating a better fit. MSE quantifies the alignment between predictions and actual outcomes.

### Design of the Recommendation System

The core function of the recommendation system is to determine the most effective treatment for individual patients. For each patient’s feature vector, the system runs all 6 RF models to predict the corresponding change in PWV. The system identifies the medication predicted to yield the most significant reduction in PWV, offering data-driven personalized treatment optimization.

This recommendation system provides a personalized approach to treatment optimization, allowing patients to benefit from the medication predicted to be most effective for them.

To provide additional insights into the recommendation system, a depth-level 10 decision tree was constructed. Tree growth was regulated using the Gini impurity index as the criterion for node splitting, stopping when the Gini index reached a value ≤20%. This decision tree serves as a valuable tool for understanding the most influential variables in the recommendation process and offers a transparent and interpretable framework.

## Results

### Characteristics of the Study Participants

The RIGIPREV study sample included a total of 194 participants with hypertension, of whom 96 (49.5%) were women. The mean age of the participants was 61.8 (SD 10.0) years. Overall, there were no differences in the baseline values of the participants included in each of the treatments except for mean corpuscular hemoglobin concentration (*P*=.02), mean corpuscular volume (MCV; *P*=.02), glycated hemoglobin (HbA_1c_; *P*=.045), and thyroid-stimulating hormone (TSH; *P*=.02). Table 1 shows the baseline characteristics of the enrolled population.

**Table 1:** Baseline characteristics of patients with hypertension from the RIGIPREV study, a multicohort analysis conducted in Spain. This table summarizes the demographic, clinical, and biochemical characteristics of participants, including age, sex, BMI, comorbidities, and medication use. The study includes individuals with hypertension recruited from the EVA, LOD-DIABETES, and EVIDENT studies, focusing on their cardiovascular health and arterial stiffness as measured by pulse wave velocity.

Characteristic				Total (N=194)		ACEI^a^ (n=44)		ARB^b^ (n=47)		BB^c^ (n=17)		Diuretic (n=14)		Diuretic+ACEI (n=28)		Diuretic+ARB (n=44)	*P* value	
**Study data, n (%)**																		
	EVA study		56 (28.9)		8 (18.2)		15 (31.9)		6 (35.3)		5 (35.7)		9 (32.1)		13 (29.5)		—^d^	
	LOD-DIABETES study		57 (29.4)		14 (31.8)		14 (29.8)		3 (17.6)		2 (14.3)		12 (42.9)		12 (27.3)		—	
	EVIDENT II study		81 (41.8)		22 (50)		18 (38.3)		8 (47.1)		7 (50)		7 (25)		19 (43.2)		—	
**Demographic factors**																		
	Age (y), mean (SD)		61.8 (10.0)		59.6 (10.0)		62.1 (8.9)		61.6 (9.5)		63.4 (10.6)		60.3 (11.9)		64.1 (9.9)		.37	
	**Gender, n (%)**																	.19
		Women	96 (49.5)		17 (38.6)		20 (42.6)		9 (52.9)		10 (71.4)		14 (50)		26 (59.1)			
		Men	98 (50.5)		27 (61.4)		27 (57.4)		8 (47.1)		4 (28.6)		14 (50)		18 (40.9)			
**Lifestyle characteristics**																		
	**Smoking, n (%)**																	.006
		Yes	26 (13.4)		10 (22.7)		7 (14.9)		1 (5.9)		0 (0)		1 (3.6)		7 (15.9)			
		No	97 (50.0)		12 (27.3)		25 (53.2)		8 (47.1)		12 (85.7)		20 (71.4)		20 (45.5)			
		Former smoker	71 (36.6)		22 (50)		15 (31.9)		8 (47.1)		2 (14.3)		7 (25)		17 (38.6)			
	Cigarette consumption (n), mean (SD)		5.75 (10.8)		8.3 (11.4)		6.7 (12.9)		5.2 (7.8)		0.7 (2.6)		2.8 (8.5)		5.7 (11.2)		.17	
	Alcohol consumption (g/wk), mean (SD)		55.5 (111.2)		47.6 (100.4)		58.3 (98.3)		38.2 (76.6)		19.2 (40.4)		51.2 (79.0)		81.4 (164.9)		.47	
	Mediterranean diet adherence (MEDAS^e^ units), mean (SD)		7.4 (2.0)		7.2 (2.1)		8.0 (1.8)		7.8 (2.6)		6.6 (1.7)		6.5 (2.2)		7.5 (2.0)		.33	
**Medication use, n (%)**																		
	**Antidiabetics**																	.19
		Yes	54 (27.8)		13 (29.5)		9 (19.1)		2 (11.8)		6 (42.9)		11 (39.3)		13 (29.5)			
		No	140 (72.2)		31 (70.5)		38 (80.9)		15 (88.2)		8 (57.1)		17 (60.7)		31 (70.5)			
	**Lipid-lowering agents**																	.1
		Yes	92 (47.4)		20 (45.5)		20 (42.6)		4 (23.5)		9 (64.3)		18 (64.3)		21 (47.7)			
		No	102 (52.6)		24 (54.5)		27 (57.4)		13 (76.5)		5 (35.7)		10 (35.7)		23 (52.3)			
	**Antiaggregants**																	.51
		Yes	43 (22.2)		10 (22.7)		15 (31.9)		3 (17.6)		2 (14.3)		6 (21.4)		7 (15.9)			
		No	151 (77.8)		34 (77.3)		32 (68.1)		14 (82.4)		12 (85.7)		22 (78.6)		37 (84.1)			
	**Anticoagulants**																	.39
		Yes	4 (2.1)		1 (2.3)		1 (2.1)		0 (0)		0 (0)		2 (7.1)		0 (0)			
		No	190 (97.9)		44 (100)		47 (100)		17 (100)		14 (100)		26 (92.9)		44 (100)			
	**NSAIDs^f^**																	.64
		Yes	1 (0.5)		0 (0)		0 (0)		0 (0)		0 (0)		0 (0)		1 (2.3)			
		No	193 (99.5)		44 (100)		47 (100)		17 (100)		14 (100)		28 (100)		43 (97.8)			
**Anthropometric measurements, mean (SD)**																		
	Height (cm)		162.5 (10.3)		164.3 (8.7)		163.5 (10.5)		159.4 (8.3)		158.5 (13.2)		163.5 (11.2)		161.4 (10.7)		.31	
	Weight (kg)		80.5 (15.3)		81.6 (13.4)		81.6 (17.0)		76.8 (12.9)		76.3 (16.1)		83.0 (17.2)		79.3 (14.6)		.63	
	Waist circumference (cm)		102.6 (10.4)		103.0 (8.5)		103.0 (12.1)		102.0 (10.8)		99.2 (10.1)		104.8 (11.9)		101.6 (9.2)		.64	
**Cardiovascular parameters**, **mean (SD)**																		
	SBP^g^ (mm Hg)		131.2 (16.4)		131.5 (13.9)		131.0 (16.4)		128.1 (18.7)		120.2 (11.9)		133.9 (18.1)		134.1 (17.1)		.11	
	DBP^h^ (mm Hg)		81.0 (11.4)		79.3 (9.8)		81.9 (9.0)		78.0 (13.0)		76.6 (7.5)		83.5 (12.9)		82.9 (14.0)		.21	
	Heart rate (beats/min)		69.7 (11.6)		69.8 (12.1)		68.6 (12.2)		63.5 (9.9)		74.2 (11.4)		70.8 (10.9)		71.2 (11.2)		.14	
	ABI^i^ (units)		1.1 (0.1)		1.0 (0.1)		1.0 (0.1)		1.1 (0.1)		1.1 (0.1)		1.1 (0.1)		1.1 (0.1)		.35	
	cfPWV^j^ (m/s)		9.5 (2.6)		8.9 (2.3)		9.5 (2.5)		9.4 (2.5)		9.9 (1.9)		9.9 (3.0)		9.8 (3.0)		.67	
	baPWV^k^ (m/s)		14.2 (2.3)		13.8 (2.3)		14.0 (2.4)		13.8 (2.0)		14.9 (2.4)		14.6 (2.3)		14.3 (2.3)		.53	
	CAVI^l^ (m/s)		8.3 (1.3)		8.0 (1.3)		8.3 (1.4)		8.0 (1.0)		8.6 (1.3)		8.6 (1.3)		8.4 (1.3)		.37	
	AIx75^m^ (%)		30.8 (10.8)		32.0 (12.5)		29.8 (10.2)		25.6 (7.9)		30.2 (13.2)		29.6 (10.4)		33.5 (9.8)		.21	
	IMT^n^ (mm)		0.7 (0.1)		0.8 (0.1)		0.7 (0.1)		0.7 (0.1)		0.6 (0.1)		0.7 (0.1)		0.7 (0.1)		.07	
	**IMT** **plaque, n (%)**																	.34
		Yes	41 (21.1)		11 (25)		11 (23.4)		3 (17.6)		2 (14.3)		3 (10.7)		11 (25)			
		No	107 (55.2)		19 (43.2)		22 (46.8)		8 (47.1)		9 (64.3)		23 (82.1)		26 (59.1)			
	Cornell index (mm), mean (SD)		15.1 (5.6)		15.2 (4.8)		15.3 (5.6)		14.2 (5.6)		14.1 (3.6)		15.1 (7.5)		15.3 (5.3)		.98	
	LVH Cornell^o^ (ms*mV), mean (SD)		1600.0 (610.6)		1499.8 (493.5)		1516.3 (610.2)		1643.9 (506.0)		1477.2 (378.0)		1662.8 (711.9)		1735.2 (700.7)		.55	
**Biochemical variables, mean (SD)**																		
	Red blood cells (million/mm^3^)		4.8 (0.4)		4.8 (0.4)		4.9 (0.4)		4.8 (0.3)		4.9 (0.6)		4.9 (0.3)		4.8 (0.4)		.87	
	Hemoglobin A (g/dL)		14.5 (1.3)		14.7 (1.3)		14.9 (1.2)		14.5 (1.0)		14.2 (1.1)		14.4 (1.5)		14.4 (1.3)		.39	
	Hematocrit (%)		43.3 (3.6)		43.9 (3.8)		44.1 (4.0)		43.4 (3.2)		42.6 (4.0)		42.9 (3.7)		42.4 (3.0)		.398	
	MCHC^p^ (g/dL)		29.7 (2.2)		30.7 (1.5)		30.2 (1.5)		30.8 (1.3)		28.1 (2.9)		29.4 (2.5)		29.1 (2.4)		*.02*	
	CHbCM^q^ (pg)		33.3 (0.9)		33.4 (0.9)		33.3 (0.8)		33.7 (0.5)		32.9 (1.3)		33.1 (1.1)		33.3 (0.8)		.61	
	MCV^r^ (fl)		89.2 (5.7)		91.7 (4.1)		90.8 (3.3)		91.6 (3.7)		85.0 (8.0)		88.9 (6.5)		87.3 (6.2)		*.02*	
	Anisocytosis index (%)		12.8 (1.2)		12.8 (1.3)		12.2 (0.4)		12.6 (0.7)		12.8 (0.6)		12.7 (1.0)		13.4 (1.7)		.07	
	Leukocytes (million/mm^3^)		6.8 (1.9)		7.1 (2.4)		7.0 (1.9)		7.0 (1.6)		7.1 (2.2)		7.1 (1.7)		6.1 (1.5)		.28	
	Neutrophils (million/mm^3^)		3.8 (1.3)		4.0 (1.8)		3.9 (1.1)		3.9 (1.2)		3.5 (1.2)		4.1 (1.2)		3.3 (0.9)		.23	
	Lymphocytes (million/mm^3^)		2.2 (0.7)		2.2 (0.6)		2.3 (1.1)		2.2 (0.6)		2.4 (0.7)		2.2 (0.6)		2.0 (0.5)		.63	
	Monocytes (million/mm^3^)		0.5 (0.1)		0.5 (0.2)		0.5 (0.1)		0.5 (0.1)		0.4 (0.1)		0.5 (0.1)		0.5 (0.1)		.79	
	Eosinophils (million/mm^3^)		0.2 (0.1)		0.1 (0.1)		0.2 (0.1)		0.2 (0.1)		0.2 (0.3)		0.1 (0.1)		0.1 (0.1)		.27	
	Basophils (million/mm^3^)		0.1 (0.1)		0.1 (0.1)		0.1 (0.0)		0.0 (0.0)		0.1 (0.2)		0.0 (0.0)		0.0 (0.0)		.59	
	Platelets (10^9^/L)		231.4 (59.5)		229.0 (66.8)		230.5 (66.5)		239.2 (60.8)		247.3 (47.3)		231.0 (60.0)		225.4 (50.4)		.92	
	RDW^s^ (%)		12.7 (0.9)		12.6 (0.8)		12.4 (0.3)		12.6 (0.5)		12.7 (0.6)		12.6 (0.7)		13.0 (1.4)		.89	
	MPV^t^ (fl)		9.4 (1.8)		10.2 (2.0)		9.0 (1.7)		9.3 (1.4)		9.5 (1.9)		8.9 (1.9)		9.6 (1.9)		.48	
	FPG^u^ (mg/100 mL)		101.7 (27.2)		102.1 (26.4)		98.0 (20.9)		90.8 (9.8)		103.2 (24.6)		113.6 (38.8)		101.3 (29.0)		.11	
	HbA_1c_^v^ (%)		5.9 (0.8)		5.8 (0.6)		5.8 (0.7)		5.5 (0.4)		6.2 (1.0)		6.2 (1.2)		5.9 (0.8)		*.045*	
	Insulin (mIU/L)		12.4 (7.6)		11.8 (6.0)		14.2 (10.5)		11.5 (5.6)		12.5 (5.4)		12.7 (6.9)		11.1 (6.6)		.49	
	Total cholesterol (mg/dL)		191.3 (32.5)		190.9 (37.2)		194.7 (28.7)		191.5 (34.5)		184.2 (23.4)		189.7 (31.9)		191.2 (34.7)		.94	
	cLDL^w^ (mg/dL)		95.9 (38.1)		89.6 (42.5)		95.5 (41.1)		97.1 (45.4)		83.3 (28.5)		104.1 (30.3)		101.2 (33.8)		.44	
	cHDL^x^ (mg/dL)		69.5 (34.3)		75.8 (42.3)		70.2 (34.7)		69.6 (33.4)		76.7 (38.1)		56.8 (21.4)		68.1 (29.9)		.33	
	Triglycerides (mg/dL)		131.0 (61.5)		135.5 (67.1)		134.6 (54.4)		123.9 (49.0)		144.5 (69.8)		145.8 (72.6)		111.7 (54.6)		.2	
	Uric acid (mg/dL)		5.7 (1.4)		5.7 (1.4)		5.8 (1.3)		5.6 (1.6)		5.8 (0.8)		5.8 (1.5)		5.8 (1.4)		.99	
	Serum creatinine (mg/dL)		0.8 (0.1)		0.8 (0.1)		0.8 (0.1)		0.8 (0.2)		0.8 (0.1)		0.8 (0.1)		0.8 (0.1)		.97	
	Creatinine (µg/min)		117.0 (66.6)		122.1 (66.8)		119.8 (65.7)		120.0 (67.1)		102.0 (63.8)		105.5 (68.7)		119.6 (68.9)		.86	
	Microalbuminuria (mg/day)		19.1 (48.7)		20.2 (52.5)		21.7 (37.7)		8.7 (10.2)		6.9 (8.2)		11.8 (18.8)		27.6 (76.6)		.596	
	Albumin/creatinine ratio		17.3 (39.0)		13.0 (28.9)		19.8 (35.4)		6.9 (7.9)		13.0 (23.0)		12.1 (25.1)		27.4 (62.2)		.55	
	eGFR^y^ (mL/min/1.73 m^2^)		85.2 (14.1)		89.2 (10.8)		85.0 (13.4)		85.8 (16.1)		81.5 (17.4)		86.0 (17.2)		82.1 (13.6)		.25	
	AST/GOT^z^ (units/L)		23.6 (12.8)		20.4 (6.1)		21.2 (5.9)		20.4 (4.2)		24.5 (7.7)		26.7 (11.0)		28.0 (22.6)		.06	
	ALT/SGPT^aa^ (units/L)		26.6 (18.5)		25.5 (14.3)		24.2 (9.7)		20.7 (8.0)		23.8 (11.3)		33.3 (25.1)		29.2 (26.6)		.18	
	GGT^a^^b^ (units/L)		32.9 (37.4)		29.5 (24.2)		30.2 (25.0)		37.3 (40.8)		22.4 (7.9)		42.8 (46.7)		34.5 (53.7)		.56	
	Fibrinogen (mg/dL)		344.0 (78.3)		345.4 (65.7)		348.1 (94.9)		359.3 (108.4)		337.9 (97.8)		350.1 (62.7)		330.2 (59.5)		.82	
	CRP^ac^ (mg/dL)		0.3 (0.4)		0.3 (0.3)		0.3 (0.5)		0.4 (0.8)		0.3 (0.3)		0.4 (0.3)		0.3 (0.4)		.95	
	TSH^a^^d^ (mU/L)		2.4 (1.3)		2.0 (1.1)		2.2 (0.9)		1.8 (1.3)		3.1 (1.2)		2.6 (1.7)		2.9 (1.6)		*.02*	
	T4^ae^ hormone (µg/dL)		1.1 (0.1)		1.1 (0.2)		1.1 (0.1)		1.1 (0.2)		1.1 (0.2)		1.2 (0.1)		1.1 (0.1)		.88	
	Vitamin D (µg/dL)		23.4 (9.7)		21.5 (9.4)		25.1 (8.0)		27.1 (11.6)		26.1 (10.7)		24.9 (10.3)		18.6 (10.0)		.46	

^a^ACEI: angiotensin-converting enzyme inhibitor.

^b^ARB: angiotensin receptor blocker.

^c^BB: β-blocker.

^d^Not available.

^e^MEDAS: Mediterranean Diet Adherence Screener.

^f^NSAID: nonsteroidal anti-inflammatory drug.

^g^SBP: systolic blood pressure.

^h^DBP: diastolic blood pressure.

^i^ABI: ankle brachial index.

^j^cfPWV: carotid-femoral pulse wave velocity.

^k^baPWV: brachial-ankle pulse wave velocity.

^l^CAVI: cardio-ankle vascular index.

^m^AIx75: augmentation index at 75 mm Hg.

^n^IMT: intima-media thickness.

^o^LVH Cornell: left ventricular hypertrophy diagnosed by Cornell.

^p^MCHC: mean corpuscular hemoglobin concentration.

^q^CHbCM: mean corpuscular hemoglobin content.

^r^MCV: mean corpuscular volume.

^s^RDW: red blood cell distribution width.

^t^MPV: mean platelet volume.

^u^FPG: fasting plasma glucose.

^v^HbA_1c_: glycated hemoglobin.

^w^cLDL: low-density lipoprotein cholesterol.

^x^cHDL: high-density lipoprotein cholesterol.

^y^eGFR: estimated glomerular filtration rate.

^z^AST/SGOT: aspartate aminotransferase/glutamic oxaloacetic transaminase.

^aa^ALT/SGPT: alanine aminotransferase/serum glutamic pyruvic transaminase.

^ab^GGT: gamma-glutamyl transferase.

^ac^CRP: C-reactive protein.

^ad^TSH: thyroid-stimulating hormone.

^ae^T4: thyroxin.

### Model Performance and Importance of Characteristics

Table 2 presents a summary of the final features used by each model in descending order of importance. The complexity of the factors influencing the effectiveness of treatments and the importance of considering a broad spectrum of clinical variables and biomarkers are shown in this table.

Specifically, the use of ACEIs was associated with the ability of weight and HbA_1c_ levels to predict the reduction in PWV. ARB, in contrast, emphasized cfPWV and low-density lipoprotein cholesterol levels as the most significant factors. BB indicated that TSH and diastolic blood pressure were the primary contributors. Diuretics exhibited high relevance for CAVI and waist circumference. In the case of diuretic+ACEI, AIx75 and uric acid were crucial factors, while diuretic+ARB were key factors for basophils and mean platelet volume. In summary, these findings underscore the intricate nature of the underlying mechanisms influencing the efficacy of various antihypertensive drugs in reducing PWV.

Furthermore, Table 3 shows the performance of the 6 predictive models used for each antihypertensive drug for both internal and external validation. The *R*^2^ values ranged from 0.61 to 0.74 in the internal validation, with the highest *R*^2^ value for the diuretic+ACEI group (*R*^2^=0.74) and the lowest for the ACEI drug group (*R*^2^=0.69). The MSE values for all drug models ranged between 0.08 for ACEIs and 0.22 for BBs. However, the model’s performance in external validation significantly decreased compared to internal validation. Thus, the *R*^2^ values ranged from 0.26 to 0.46, and the MSE values varied from 0.29 to 0.45.

**Table 2:** Most significant features contributing to the regression models evaluating the efficacy of each antihypertensive medication in the RIGIPREV study. This table highlights the key features that provide the most information for each regression model, which represents the efficacy of different antihypertensive drugs, including angiotensin-converting enzyme inhibitors (ACEIs), angiotensin receptor blockers (ARBs), β-blockers (BB), diuretics (DIU), and combinations.

Importance	ACEI	ARB	BB	DIU	DIU+ACEI	DIU+ARB
1	Weight	cfPWV^a^	TSH^b^	CAVI^c^	AIx75^d^	Basophils
2	HbA_1c_^e^	cLDL^f^	DBP^g^	Waist circumference	Uric acid	MPV^h^
3	ALT/SGPT^i^	AST/GOT^j^	cfPWV	Vitamin D	ABI^k^	MCV^l^
4	Creatinine	CHbCM^m^	HbA_1c_	cHDL^n^	LVH Cornell^o^	LVH Cornell
5	baPWV^p^	Alx75	Albumin and creatinine	cLDL	HbA_1c_	DBP
6	Hemoglobin	Uric acid	cHDL	MPV	Triglycerides	Hematocrit
7	LVH Cornell	FPG^q^	Uric acid	Creatinine	cfPWV	TSH
8	cHDL	Total cholesterol	Triglycerides	RDW^r^	Height	cfPWV
9	Uric acid	GGT^s^	ABI	IMT^t^ plaque	GGT	Monocytes
10	Red blood cell distribution width	eGFR^u^	eGFR	IMT	baPWV	IMT

^a^cfPWV: carotid-femoral pulse wave velocity.

^b^TSH: thyroid-stimulating hormone.

^c^CAVI: cardio-ankle vascular index.

^d^AIx75: augmentation index at 75 mm Hg.

^e^HbA_1c_: glycated hemoglobin.

^f^cLDL: low-density lipoprotein cholesterol.

^g^DBP: diastolic blood pressure.

^h^MPV: mean platelet volume.

^i^ALT/SGPT: alanine aminotransferase/serum glutamic pyruvic transaminase.

^j^AST/GOT: aspartate aminotransferase/glutamic oxaloacetic transaminase.

^k^ABI: ankle-brachial index.

^l^MCV: mean corpuscular volume.

^m^CHbCM: mean corpuscular hemoglobin content.

^n^cHDL: high-density lipoprotein cholesterol.

^o^LVH Cornell: left ventricular hypertrophy diagnosed by Cornell.

^p^baPWV: brachial-ankle pulse wave velocity.

^q^FPG: fasting plasma glucose.

^r^RDW: red blood cell distribution width.

^s^GGT: gamma-glutamyl transferase.

^t^IMT: intima-media thickness.

^u^eGFR: estimated glomerular filtration rate.

**Table 3:** Performance evaluation of predictive models by medication using R2 and mean squared error (MSE) with internal and external validation. This table summarizes the performance of machine learning models predicting the impact of antihypertensive drugs on pulse wave velocity in patients with hypertension. Results are based on R² and mean square error for internal and external validation using data from the RIGIPREV study cohorts in Spain.

Drug	Internal validation			External validation	
	*R* ^2^	MSE	*R* ^2^		MSE
ACEI^a^	0.69	0.08	0.26		0.31
ARB^b^	0.72	0.09	0.42		0.38
BB^c^	0.61	0.22	0.31		0.45
Diuretic	0.71	0.12	0.40		0.29
Diuretic+ACEI	0.74	0.15	0.46		0.33
Diuretic+ARB	0.70	0.15	0.29		0.32

^a^ACEI: angiotensin-converting enzyme inhibitor.

^b^ARB: angiotensin receptor blocker.

^c^BB: β-blocker.

### Analysis of Medication Recommendations

**Figure 1:**
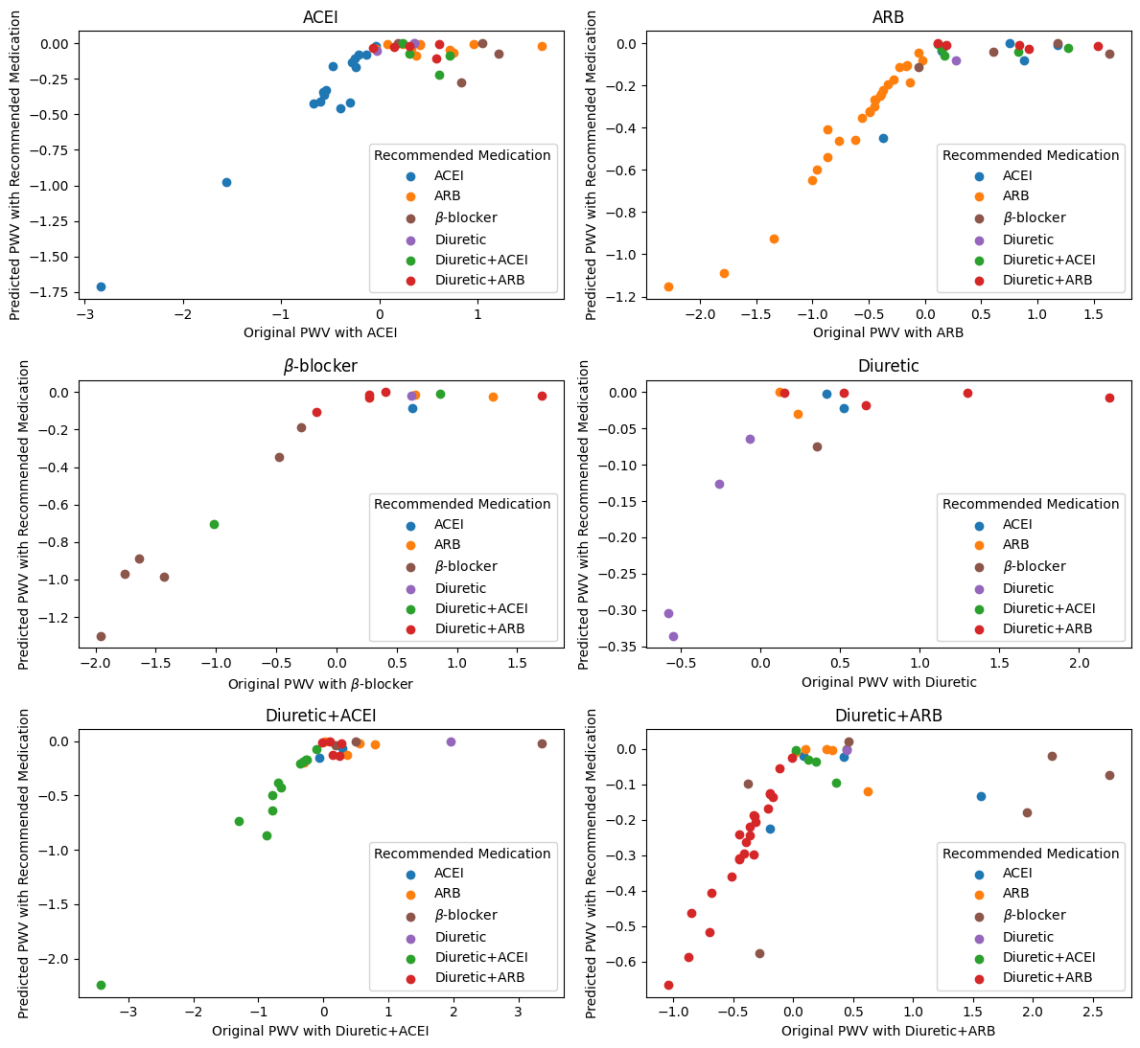
Comparison of original versus predicted pulse wave velocity (PWV) in patients with hypertension. This figure shows the comparison between the original PWV values and those predicted by the recommendation system in patients with hypertension from the RIGIPREV study. The model suggests optimized antihypertensive treatments based on individual patient characteristics. ACEI: angiotensin-converting enzyme inhibitor; ARB: angiotensin receptor blocker.

Figure 1 shows the distribution of patient recommendations by medication. There was substantial variability in medication recommendations compared to the original antihypertensive drugs taken by patients. ARB medication was the most frequently recommended, appearing in 26.8% (52/194) of the patients, followed by ACEI, which was recommended in 15.5% (30/194) of the patients. Notably, of the patients who were originally on ACEIs, the same recommendation (match rate) was made for only 36% (16/44) of the patients. For patients who were originally on ARBs, a substantially greater match rate of 55% (26/47) was observed. The combination antihypertensive drugs such as diuretics+ARB and diuretics+ACEI, 52% (23/44) and 43% (12/28), respectively, maintained the same combination medication regimens.

### Modeling Medication Recommendations With a Decision Tree

**Figure 2:**
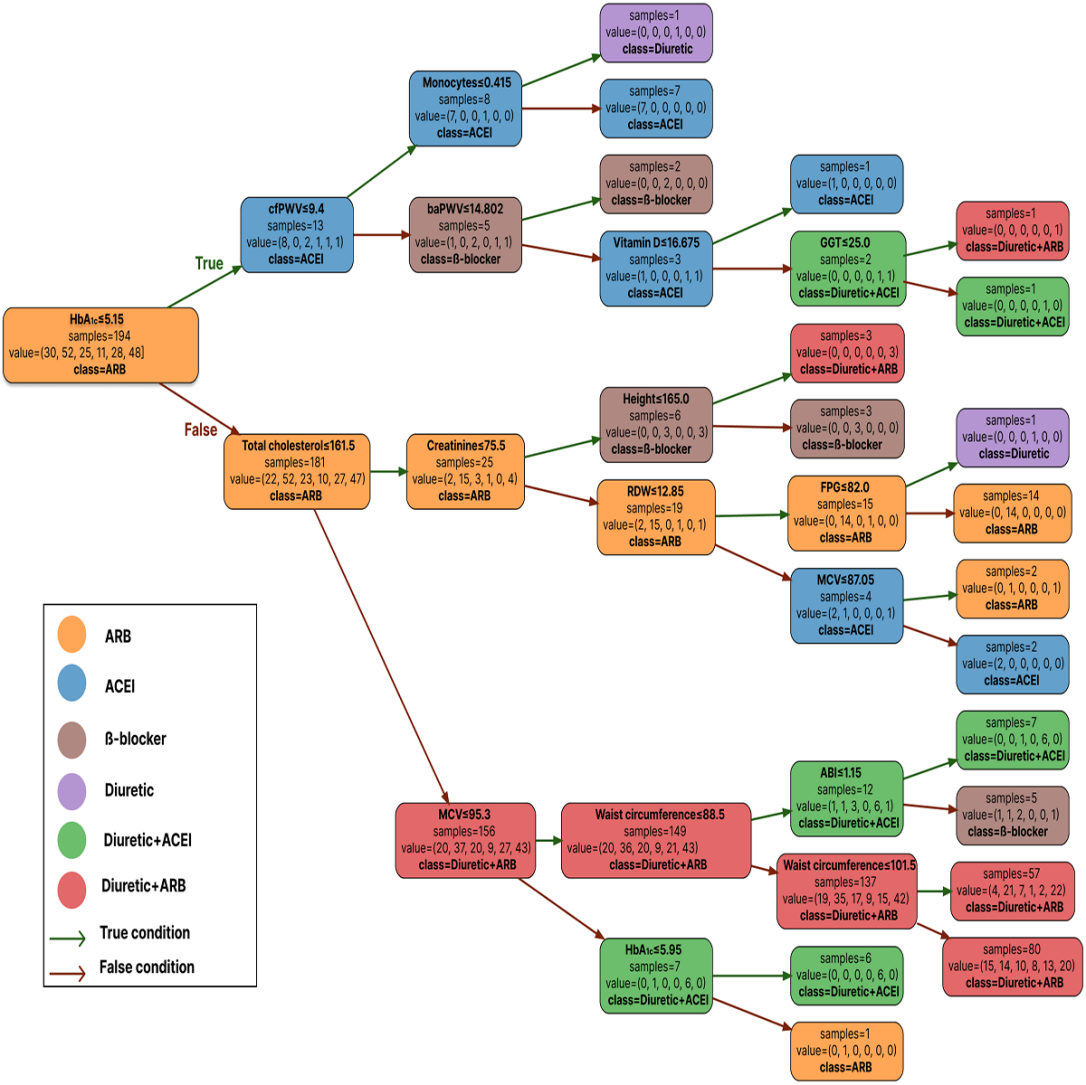
Visual decision tree representation of the underlying mechanisms and workflow of the recommendation system. This decision tree visualizes the workflow of the machine learning–based recommendation system, showing key predictors such as baseline pulse wave velocity (PWV) and patient characteristics. It was developed using data from the RIGIPREV study, conducted on patients with hypertension in Spain. ABI: ankle-brachial index; ACEI: angiotensin-converting enzyme inhibitor; AIx75: augmentation index at 75 mm Hg; ALT/SGPT: alanine aminotransferase/serum glutamic pyruvic transaminase; ARB: angiotensin receptor blocker; AST/SGOT: aspartate aminotransferase/glutamic oxaloacetic transaminase; baPWV: brachial-ankle PWV; BB: β-blocker; CAVI: cardio-ankle vascular index; cfPWV: carotid-femoral PWV; cHDL: high-density lipoprotein cholesterol; cLDL: low-density lipoprotein cholesterol; CRP: C-reactive protein; DBP: diastolic blood pressure; eGFR: estimated glomerular filtration rate; FPG: fasting plasma glucose; GGT: gamma-glutamyl transferase; HbA1c: glycated hemoglobin; IMT: intima-media thickness; LVH Cornell: left ventricular hypertrophy diagnosed by Cornell; MCV: mean corpuscular volume; MPV: mean platelet volume; RDW: red blood cell distribution width; TSH: thyroid-stimulating hormone.

Figure 2 shows the decision tree for selecting a suitable antihypertensive drug for PWV reduction based on patient characteristics. An overall accuracy of 84.02% was achieved when the decision tree was fitted to the medication recommendations generated by the original RF models, with 10 levels of depth. However, in terms of interpretability, only 5 levels are shown in Figure 2. After examination, the decision tree revealed that ARBs and ACEIs were the first antihypertensive drug options based on HbA_1c_. At the second level, based on baseline cfPWV levels when a person is taking ACEI, the antihypertensive drug should be BBs or maintain ACEI, and based on total cholesterol when a person is taking ARA, diuretic+RA, or maintain ARA. Diuretics and diuretics+ACEI did not appear until the third level based on monocytes and MCV, respectively, and depended on the levels of the previous characteristics.

## Discussion

### Effectiveness of the Predictive Models and Validation

In this study, we developed models using embedded RF ML models for multitarget prediction to provide recommendations on the most suitable antihypertensive drug for reducing PWV, based on the individual characteristics of participants with hypertension. The predictive models used demonstrated a high level of fit between the predictions and validation data, with *R*^2^ values of approximately 0.65 for all the models in the internal validation. In addition, they exhibit low MSEs ranging from 0.08 to 0.22, indicating that the model predictions significantly align with the PWV values.

However, the performance of the model significantly decreased in external validation compared to its performance in internal validation. The *R*^2^ value decreased by approximately 0.33 on average (SD 0.06), while the MSE increased by an average of 0.21 (SD 0.05). Although this decrease could indicate potential overfitting of the regression models to the training data, it is important to consider that the use of SMOTE for generating training data could also play a role in this overfitting. The synthetic augmentation of data, while valuable for addressing sample size limitations, may introduce patterns or characteristics that are not present in the original dataset, potentially leading to models that are overly optimized for the synthetically enhanced training set rather than real-world data. This phenomenon underscores the complex balance between enhancing the dataset size for model robustness and ensuring that such augmentation accurately reflects the true data distributions.

Nevertheless, the results suggest that the models can explain a significant portion of the variance in PWV prediction and, consequently, affirm their reliability in providing recommendations in clinical practice.

### Importance of Variables and Medication Recommendations

The intrinsic capability of RFs to evaluate and prioritize the importance of input features during training plays a pivotal role in understanding which variables crucially influence the predictions. This aligns seamlessly with our objective to guide clinicians in selecting the most appropriate medication by identifying key determinants of treatment effectiveness. Unlike other models, such as support vector machines, which might offer precision but lack the ability to inherently track feature importance, RF models provide unique insights into how specific variables impact outcomes, making them particularly valuable in scenarios where understanding these impacts is as crucial as achieving high prediction accuracy.

The variable weights for each of the 6 antihypertensive drug models for PWV reduction differed: ACEIs were associated with weight and HbA_1c_, ARBs with cfPWV and low-density lipoprotein cholesterol, BBs with TSH and DBP, diuretics with CAVI and waist circumference, diuretic+ACEI with AIx75 and uric acid, and diuretic+ARB indicated basophils and mean platelet volume levels as the most important factors. Similarly, we found significant variability in the recommendations of the different models regarding the antihypertensive drug that most effectively reduced the PWV in each participant compared to the original antihypertensive drugs taken by the patients, with a matching rate of 55.3% for ARBs. Finally, the decision tree for selecting the most suitable antihypertensive drug to reduce PWV based on patient characteristics revealed that, at the first level, ARBs and ACEIs were the top choices based on HbA_1c_, and at the second level, based on baseline cfPWV for ACEIs, the antihypertensive drug should be either BBs or continuation of ACEIs, while based on total cholesterol levels for ARBs, it should be diuretic+ARB or ARB alone.

### Advantages of RF Models in the Clinical Context

RF models can handle a wide variety of variable types (both continuous and discrete) within their decision-making processes, effectively capturing the nonlinear relationships among variables. This is a significant advantage over linear models, which may struggle to interpret these complex relationships without prior data transformation. In addition, the ability of RF to manage high-dimensional data by identifying and selecting the most relevant features for model training (thereby reducing dimensionality without the need for manual intervention) distinguishes it from models such as k-nearest neighbors, which can suffer performance issues in high-dimensional spaces.

Currently, numerous clinical practice guidelines establish common guidelines for hypertension treatment and emphasize the importance of making a personalized choice regarding the most suitable antihypertensive drug [3,4]. However, the medical approach to choosing an antihypertensive drug is based on its ability to reduce blood pressure, individual patient needs, and potential side effects [3,4], without considering the effect of each antihypertensive drug on AS, although AS is an independent risk factor for morbidity and mortality from cardiovascular disease [5,6]. Therefore, we must choose an antihypertensive drug that considers both lowering blood pressure and reducing AS based on the individual characteristics of the participant. However, currently, physicians do not have sufficient information to consider all these aspects when making decisions. After demonstrating the beneficial effect [8-11] of antihypertensive drugs on AS, we analyzed individual characteristics to predict a greater reduction in AS for each antihypertensive drug using ML techniques to identify sophisticated patterns by analyzing each patient’s variables and thus improving personalized care [21]. Furthermore, the embedded RF ML model used in this study is an integrated learning approach that offers excellent prediction capabilities and is tolerant of outliers [46].

Indeed, the essence of our prediction system lies precisely in its ability to infer potential outcomes for a patient based on information from other patients with similar characteristics who have been treated with various medications. In this regard, while a patient who has taken only one type of medication during the data collection period provides a direct outcome for that specific medication, the recommendation system uses 6 predictive models that have been trained with all patients who have taken different medications. Therefore, this learning process enables the system to predict how other medications could have impacted the patient by considering how patients with similar profiles responded to those other treatment options. For instance, by comparing these covariates and the observed outcomes in the dataset, the system can suggest that a patient who took ACEIs, for example, might benefit more from ARBs, based on patterns identified in patients with similar characteristics.

### Comparison With Previous Studies

In recent years, numerous studies have examined ML techniques to assess which antihypertensive drug is most suitable for controlling blood pressure as well as the variables with the most significant weight in indicating its use, but we have not found studies that analyzed the effect on PWV. Among these studies, we aimed to construct a prediction model for antihypertensive drugs to be used in older patients with hypertension to help physicians quickly and rationally combine the most appropriate antihypertensive drugs based on their individual characteristics. The authors compared 5 models and found that the Light Gradient Boosting Machine algorithm predicted the best results, identifying different variables for each antihypertensive drug analyzed [27]. Koren et al [47] applied ML techniques such as decision trees and neural networks to determine how to treat hypertension in a large group of patients from the Maccabi Healthcare Service in Israel. They found that the number of patients with hypertension treated with a single antihypertensive drug was greater than the number of patients with hypertension treated with combination therapy, and the monotherapy control rate was greater than the success rate of 2-, 3-, or 4-drug combinations. Liu et al [26] profiled 5 commonly used antihypertensive drugs (irbesartan, metoprolol, felodipine, amlodipine, and levamlodipine) and provided data for the customization of clinical antihypertensive drugs. All these studies could help tailor the administration of effective antihypertensive drugs to individual patients. Our study goes a step further and provides information on the most effective antihypertensive drugs for reducing AS on an individualized basis.

### Conclusions and Limitations of This Study

In summary, this study included participants with hypertension on antihypertensive treatment from 3 research projects with at least 2 PWV measurements and a wide range of variables who were followed for at least 1 year. We developed individual recommendations to determine which antihypertensive drug achieves the greatest reductions in PWV based on individual characteristics. These results can assist physicians in making decisions regarding the prescription of antihypertensive drugs considering not only blood pressure reduction but also PWV reduction. The analysis also underscores the importance of a personalized approach to drug selection, considering a broad spectrum of patient characteristics. The differences in indicators found among antihypertensive drug groups will help guide our selection of antihypertensive drugs.

This study has several limitations, such as the omission of variables that have been shown to influence blood pressure levels, such as dietary salt intake [48], and the inclusion of variables that are not routinely measured in clinical practice, such as insulin levels. Consequently, the dataset considered for the investigated clinical question may not be the most appropriate, and the sample size is small. Nevertheless, the study also has strengths, as the data come from records of studies conducted in various research projects, and the measurement of variables was uniform and protocolized, a guarantee that cannot be provided when using electronic health record data. Given that these models learn from examples, data quality is fundamental; without meticulously selected and labeled data, models cannot be effectively built or evaluated. Furthermore, the participants who were treated with each antihypertensive drug were not randomized, but they were selected at the discretion of the clinician, which may bias our results because, although there were no statistically significant differences in most of the baseline variables, there were significant differences in mean corpuscular hemoglobin concentration, MCV, HbA_1c_, and TSH. Finally, the main limitation of this study is related to the sample size. Although a train-test split of 80%-20% was used to evaluate the model’s performance, both the training and test samples originated from the same databases. Consequently, the generalizability of the model to entirely new and external datasets remains untested. Ideally, in line with the Transparent Reporting of a multivariable prediction model for Individual Prognosis or Diagnosis guidelines [49], an external validation using a completely independent dataset would have been performed to robustly ascertain the model’s applicability across various patient populations. While the current approach provided valuable insights, a broader sample base in future studies could offer an even more robust understanding of the model’s applicability across different patient groups.

Our research bridges a crucial gap in the current management of hypertension by not only prioritizing the reduction of blood pressure but also considering the effect on AS, as measured by PWV. AS is a widely accepted independent predictor of cardiovascular diseases. The importance of this lies in the customization of antihypertensive drug recommendations that not only effectively lower blood pressure but also aid in decreasing AS, thereby potentially reducing the overall risk of cardiovascular disease.

Our findings hold significant importance, and this can be better explained through the points mentioned subsequently. First, our study addresses a critical gap in current hypertension management by not only focusing on blood pressure reduction but also considering the impact on AS, as measured by PWV. AS is a well-established independent risk factor for cardiovascular diseases. The significance lies in tailoring antihypertensive drug recommendations that not only effectively lower blood pressure but also contribute to reducing AS, thus potentially lowering overall cardiovascular risk. Second, the personalized recommendations generated by our models offer a patient-centric approach to antihypertensive therapy. By considering individual characteristics and responses, clinicians can make more informed decisions, potentially enhancing treatment adherence and overall patient outcomes. Third, the use of advanced ML techniques, specifically the embedded RF model, enhances the reliability and sophistication of our recommendations. ML methods, when integrated appropriately, have the potential to reveal intricate patterns within complex datasets, providing more nuanced and accurate predictions than traditional approaches. Fourth, while the primary focus is on antihypertensive recommendations, it is essential to highlight that our study integrates evidence from 3 distinct studies (the EVA study, the LOD-DIABETES study, and the EVIDENT study), each with the following unique protocols. The careful consideration of key variables, consistent measurement of PWV, and adjustment for various covariates strengthen the validity of our results. In addition, the model performance metrics, including *R*^2^ values and MSEs, indicate the robustness of our predictive models.

Furthermore, before the conclusions of this study, it is important to examine future work and the limitations of our study. Our analysis is primarily focused on data gathered at the initial and subsequent follow-up points. However, as we recognize the fluid nature of patient well-being, we are keen to examine the potential impact of incorporating multiple follow-up sessions within the data-gathering phase. This involves treating patients as dynamic variables during each follow-up, providing a more comprehensive understanding of their progressing health patterns. As we delve into our future work, addressing potential transitions between antihypertensive medications for patients during the follow-up period holds great importance. To enhance our recommendation system, we delve into the implications of these changes and consider effective methodologies to account for them. It is imperative that we understand the interplay between patient characteristics, medication choices, and health outcomes. To enhance the strength of our recommendations, we will delve into the potential for causal inference within our model and explore the incorporation of supplementary outcomes.

In conclusion, in this study, we developed predictive models to provide recommendations regarding the most suitable antihypertensive drug for reducing PWV based on the individual characteristics of patients with hypertension. We found that at the first level, ARBs and ACEIs were the top choices based on HbA_1c_, and at the second level, based on baseline cfPWV levels for ACEIs, antihypertensive drugs should be either BBs or ACEIs, while based on total cholesterol levels for ARBs, they should be diuretics+ARBs or ARBs alone. Therefore, our study provides a data-based reference for the customization of antihypertensive drugs to reduce PWV.

## Abbreviations

ACEI: angiotensin-converting enzyme inhibitor
ARB: angiotensin receptor blocker
AS: arterial stiffness
baPWV: brachial-ankle pulse wave velocity
BB: β-blocker
CAVI: carotid-artery vascular index
CCB: calcium channel blocker
cfPWV: carotid-femoral pulse wave velocity
HbA1c: glycated hemoglobin
MCV: mean corpuscular volume
ML: machine learning
MSE: mean squared error
PWV: pulse wave velocity
RF: random forest
SMOTE: Synthetic Minority Oversampling Technique
TSH: thyroid-stimulating hormone

## Data Availability

The datasets generated during and analyzed during this study are available from the corresponding author on reasonable request. In addition, the code used to develop the machine learning models and recommendation system will be made publicly available through Mendeley library.
